# Long-term quality of life in head and neck cancer: the role of postdiagnosis smoking behavior

**DOI:** 10.1007/s11764-025-01894-2

**Published:** 2025-10-21

**Authors:** Elham Mohebbi, Alaina L. Carr, Garret Guthrie, Siddhi Patil, Emily Clementi, Jonathan Giurintano, Matthew Witek, Ming Tan, Ruzong Fan, Steven Chinn, Gregory Wolf, Kristi D. Graves, Peter H. Ahn, Laura Rozek

**Affiliations:** 1 Department of Oncology, School of Medicine, Georgetown University, Washington, DC USA; 2 Department of Biostatistics, Bioinformatics and Biomathematics, Georgetown University Medical Center, Washington, DC USA; 3 School of Medicine, Georgetown University, Washington, DC USA; 4 Department of Otolaryngology, MedStar Georgetown University Hospital, Washington, DC USA; 5 Department of Otolaryngology–Head and Neck Surgery, University of Michigan, Ann Arbor, MI USA; 6 Department of Radiation Medicine, MedStar Georgetown University Hospital, Washington, DC USA; 7 Cancer Prevention and Control Program, Lombardi Comprehensive Cancer Center, 2115Wisconsin Ave NW, Washington, DC 20007 USA

**Keywords:** Quality of life, Head and neck neoplasms, Mouth neoplasms, Smoking cessation, Psychological well-being, SF-36

## Abstract

**Purpose:**

Head and neck squamous cell carcinoma (HNSCC) significantly impacts patients’ quality of life (QoL). This study investigated long-term QoL outcomes among 619 patients with newly diagnosed HNSCC over five years.

**Methods:**

QoL was assessed annually using the SF-36, covering physical functioning, emotional well-being, social functioning, pain, and general health. Patients were stratified by tumor subsite and smoking status.

**Results:**

Significant improvements in QoL were observed at the 1-year follow-up, particularly among patients with pharyngeal [adjusted β: 3.56 (95% CI: 1.69, 5.43)] and oral cavity cancers [adjusted β: 2.56 (95% CI: 0.52, 4.60)]. However, patients with laryngeal cancer showed no significant change at any follow-up [e.g., 1-year adjusted β: 0.93 (95% CI: -2.36, 4.24)]. Overall, QoL improved most notably in the early years post-treatment [adjusted β: 2.86 (95% CI: 1.56, 4.16)]. Smoking status significantly influenced QoL trajectories. Never-smokers showed the greatest improvement (from 53.11 at baseline to 59.29 at year 2, P < 0.0001), followed by quitters (48.09 to 51.42, P = 0.04). Continued and intermittent smokers demonstrated no significant gains.

**Conclusions:**

In this longitudinal study of HNSCC patients, quality of life significantly improved over time, particularly among those with pharyngeal and oral cavity tumors and those who never smoked or quit smoking after diagnosis.

**Implications for Cancer Survivors:**

These findings highlight the importance of smoking cessation in survivorship care. Personalized care strategies addressing both physical and psychosocial challenges, particularly for continued smokers and those with laryngeal tumors, are essential to improving long-term QoL in HNSCC survivors.

**Supplementary Information:**

The online version contains supplementary material available at https://doi.org/10.1007/s11764-025-01894-2.

## Introduction

Head and neck squamous cell carcinoma (HNSCC) is a prevalent malignancy associated with significant morbidity and mortality [1]. Treatment for HNSCC often involves a combination of surgery, radiation, and chemotherapy, which can lead to substantial functional impairments and a poor quality of life (QoL) in patients. As survival rates for HNSCC continue to improve, understanding the long-term impact of treatment on QoL has become increasingly important [2]. Numerous studies have shown that patients with HNSCC often experience declines in multiple QoL domains, including physical functioning, social functioning, pain, and emotional well-being, particularly in the immediate years following treatment [3, 4]. However, it is crucial to examine how these changes in QoL evolve over time, significantly beyond the first few years post-treatment.

Several factors contribute to variations in QoL outcomes among HNSCC patients, including demographic characteristics, tumor subsites, and treatment modalities. For instance, patients with pharyngeal cancers have reported better recovery and QoL scores than those with laryngeal or oral cavity tumors, with tumor subsite playing a crucial role in shaping the patient's overall recovery trajectory [5–8]. Additionally, smoking status has been identified as a significant determinant of QoL in HNSCC patients. Specifically, smoking cessation post-diagnosis is associated with improved QoL, while continued smoking or intermittent smoking patterns negatively impact health outcomes and recovery [9, 10]. The role of smoking, comorbidities, and cancer stage at diagnosis is a critical factor influencing both the physical and psychosocial aspects of QoL [11, 12].

This study investigated the long-term QoL outcomes in 619 patients with newly diagnosed HNSCC over five years, focusing on the impact of demographic and clinicopathologic factors, such as cancer subsite, post-diagnosis smoking status, and treatment modality. Specifically, we examined how QoL scores changed over time across different HNSCC subsites, emphasizing the role of smoking cessation and its effects on physical and emotional health domains. Understanding these factors could provide valuable insights into improving personalized care strategies and guiding future interventions for HNSCC patients in the long-term survivorship phase.

## Materials and methods

### Study design and population

This prospective cohort study was conducted with 619 newly diagnosed patients in the past 12 months, diagnosed with HNSCC, recruited from the University of Michigan Hospital between 2008 and 2014. While the study follow-up period extended up to eight years, most patients did not yet reach years 5–8 at the time of data collection. As a result, the number of observations in those later years was limited. For this reason, we focused our main analyses on the first five years. Patients were excluded if they were younger than 18 years of age, pregnant, non-English speakers, had mental health concerns, or had other non-upper aerodigestive tract tumors or additional primary HNSCC diagnoses in the last 5 years.

### Quality of life assessment

At baseline, demographic characteristics, including gender, age, and clinical features such as cancer subsite, cancer stage, comorbidities, and Body Mass Index (BMI), were collected. Data for this analysis were obtained prospectively through mailed questionnaires, with phone calls used only to support participant completion of follow-up surveys and medical records and physician input to ensure data completeness over the eight-year period. Quality of life was assessed using the SF-36 [13]. This widely used instrument evaluates patients' perceived health across eight domains: physical functioning (PF), role limitations due to physical health (RP), role limitations due to emotional problems (RE), energy/fatigue (EF), emotional well-being (WE), social functioning (SF-Social), pain (P), and general health (GH). The SF-36 is a 36-item questionnaire that asks participants to rate their health in terms of the difficulty they experience in each of the eight domains over a specified period. Each domain is scored on a scale of 0–100, with higher scores indicating better health.

The SF-36 has been widely validated across multiple populations and is considered a robust tool for evaluating both general and specific health status in chronic illnesses, including HNSCC [14, 15]. It provides a comprehensive assessment of physical, emotional, and social health. The questionnaire was mailed to patients at baseline and at each follow-up point to measure the changes in QoL over time.

Each patient was asked to complete the SF-36 questionnaire, and their responses were scored using the standardized scoring protocol [16]. The responses for each domain were summed, with higher scores indicating better functioning and fewer limitations. These domain scores were then aggregated to calculate the overall QoL score, which provides a comprehensive measure of a patient's perceived health status. Patients' QoL scores were stratified by HNSCC subsites. We used the cleaned and finalized dataset, excluding patients with missing data at any follow-up time point. These factors were examined to explore their impact on QoL across the different time points, as well as to identify potential predictors of long-term health outcomes. A difference of 4–5 points on the SF-36 has been reported as the minimal clinically important difference (MCID) in several populations, including adult patients with cancer and chronic conditions [17–19]. Although thresholds may vary slightly by domain and population, a commonly accepted approximation is half of a standard deviation, or approximately 5 points for SF-36 T-scores standardized to a mean of 50 and SD of 10 [20, 21]. This study adopts this threshold to interpret the clinical relevance of observed QoL differences among head and neck cancer survivors. While statistical significance was assessed in the analysis, observed differences that did not meet a clinically significant threshold should be interpreted with caution, as they may not reflect clinically meaningful changes [16].

### Measures

The participants were filled out a structured questionnaire that gathered detailed information on demographics, behavioral factors, exposures, and health conditions. A comprehensive smoking history was collected, including the duration and intensity of tobacco use, as well as the average number of tobacco products consumed daily. Using this information, the pack-years of tobacco smoking were calculated. All current smokers were offered a referral to a smoking cessation clinic as part of the study. Based on their smoking history, participants were categorized into four groups: never-smokers (*N* = 162), who had never smoked; former smokers (*N* = 81), who quit more than one year before diagnosis; quitters (*N* = 145), who stopped within a year prior to diagnosis and post-diagnosis; continuing smokers (*N* = 103), who continued smoking after pre- and post-diagnosis; and intermittent smokers (*N* = 128), who quit more than a year before diagnosis but had smoked at least once after diagnosis.

Additional potential confounders were also collected, such as alcohol consumption, which was documented in the same manner as tobacco smoking. The diagnosis date was determined based on the day the patient received a biopsy-confirmed diagnosis of squamous cell carcinoma at the University of Michigan. Comorbidities were assessed at diagnosis using the Adult Comorbidity Evaluation-27 (ACE-27), a validated tool for evaluating comorbidity severity in cancer patients [22]. For this study, HNSCC was classified as a new diagnosis if no primary tumor had been identified within the previous five years.

A team of local experts reviewed all relevant medical documents and completed a questionnaire that included clinical and histopathologic features of the tumor, as well as any existing illnesses. Treatment modalities were standardized and discussed by our multidisciplinary expert tumor board. No significant changes occurred in diagnostic or treatment protocols during the study period. Tumor staging was classified based on the 7th edition of the TNM classification system, as documented in the medical records prior to any treatment.

### Statistical analysis

Descriptive statistics were used to summarize the demographic and clinical characteristics of the study participants. Continuous variables were summarized as means ± standard deviations (SD), and categorical variables were expressed as frequencies and percentages. We employed multiple linear regression models to compare QoL scores across different HNSCC subsites. These models were adjusted for potential confounders (See confounders below). This adjustment allowed us to isolate the effects of each factor on QoL while controlling for other variables that could potentially influence outcomes. We estimated adjusted beta coefficients (β) for each variable. These coefficients represent the direction of the association between each factor and the QoL outcome. We reported the 95% confidence intervals (CIs) for each regression analysis for the adjusted beta coefficients and the p-values for testing if the coefficients are zero. The confidence intervals assess the estimates' precision, with intervals that do not contain 0 indicating statistically significant associations. Statistical analyses were conducted using SAS version 9.4.

We explored the option of imputing missing values of questionnaire items by carrying forward the last observed score within each participant across time points. This approach, commonly used in longitudinal data analysis to address dropout or intermittent missingness, was implemented using the last observation carried forward (LOCF) method [23]. After conducting parallel analyses with the imputed dataset, we found no meaningful difference in the direction, magnitude, or statistical significance of the results compared to the original dataset. Therefore, to preserve the integrity of the observed data and avoid potential biases introduced by imputation, we opted to retain the original, unimputed dataset for all final analyses.

### Confounders

To control for potential confounding, we included several demographic and clinical variables in our models [24–26]. These confounders were selected based on prior literature and clinical relevance and included age at diagnosis, gender, BMI, cancer subsite, cancer stage (based on TNM 7th edition), comorbidity score (measured using the Adult Comorbidity Evaluation-27), treatment modality (surgery, radiation, chemotherapy, or combination), smoking status (never, former, quitters, continued, intermittent), and alcohol consumption (never, former, current). These variables were collected at baseline, and all follow up time points, and included in all regression models to isolate the effects of key exposures on QoL outcomes.

## Results

### Participant characteristics and baseline QoL

Table 1 presents the demographic and clinicopathologic characteristics of 619 HNSCC patients and their baseline QoL scores. Among the participants, 458 (73.99%) were men, and 161 (26.01%) were women. Current smoking was most common among patients with laryngeal cancer (67.6%, 102/151), followed by those with oral cavity cancer (50.6%, 160/316) and pharyngeal cancer (37.5%, 138/368). The mean age of participants was 60.88 ± 11.37 years, with a median age of 60. Patients aged ≤ 60 years had a baseline mean QoL score of 51.43 (95% CI: 50.17, 52.69), while those aged > 60 years had a lower mean QoL score of 48.54 (95% CI: 47.22, 49.85) at baseline (*P* = 0.001). Men had much better QoL at baseline than women (*P* < 0.0001). Regarding cancer subsite, most patients had tumors in the pharynx (46.20%), followed by the oral cavity (36.51%) and the larynx (17.29%). The mean QoL score at baseline was highest in patients with pharyngeal cancer, 51.68 (95% CI: 50.32, 53.04), following oral cavity cancer, 49.27 (95% CI: 47.83, 50.70) and lowest in those with laryngeal cancer, 47.42 (95% CI: 45.12, 49.72) (*P* = 0.002) (Table 1).

**Table 1:** Demographic and clinicopathologic characteristics of 619 head and neck cancer patients

	Frequency (%)	QoL score at baseline (95% CI)	*P*-value
Total	619		
Gender			
Men	458 (73.99)	51.16 (50.12, 52.20)	< 0.0001
Women	161 (26.01)	46.94 (45.11, 48.77)	
Age (25–95, Median = 60)	60.88 ± 11.37		
≤ 60	327	51.43 (50.17, 52.69)	0.001
> 60	292	48.54 (47.22, 49.85)	
Tumor subsite			
Oral cavity	226 (36.51)	49.27 (47.83, 50.70)	0.002
Pharynx	286 (46.20)	51.68 (50.32, 53.04)	
Larynx	107 (17.29)	47.42 (45.12, 49.72)	
Stage			
In situ	10 (1.62)	67.50 (57.19, 77.80)	0.030
I	95 (15.35)	60 (54.97, 65.02)	
II	78 (12.60)	54.11 (48.68, 59.54)	
III	83 (13.41)	56.39 (51.04, 61.75)	
IV	353 (57.03)	54.81 (52.31, 57.32)	
BMI			
Underweight	22 (3.55)	44.30 (38.56, 50.03)	0.010
Normal	190 (30.69)	48.76 (47.09, 50.43)	
Overweight	231 (37.32)	51.44 (49.97, 52.91)	
Obese	176 (28.43)	50.39 (48.70, 52.08)	
Comorbidity			
None	159 (25.69)	54.02 (52.35, 55.69)	< 0.0001
Mild	306 (49.43)	46.66 (48.34, 50.99)	
Moderate	114 (18.42)	47.80 (45.84, 49.76)	
Severe	40 (6.46)	43.85 (40.09, 47.61)	
Treatment			
Chemoradiation therapy	274 (44.26)	51.03 (49.59, 52.46)	0.037
Radiation therapy	43 (6.95)	50.47 (46.84, 54.10)	
Surgery	158 (25.53)	50.68 (48.97, 52.38)	
Surgery + Adjuvant radiation therapy	66 (10.66)	46.93 (44.38, 49.48)	
Surgery + Adjuvant chemoradiation therapy	78 (12.60)	48.03 (45.38, 50.67)	
Smoking			
Never	164 (26.49)	53.16 (51.55, 54.78)	< 0.0001
Current	248 (40.06)	46.88 (45.39, 48.37)	
Former	207 (33.44)	51.42 (49.89, 52.94)	
Alcohol drinking			
Never	35 (5.65)	49.01 (45.27, 52.75)	0.001
Current	362 (58.48)	51.47 (50.30, 52.65)	
Former	222 (35.86)	47.93 (46.37, 49.48)	

### QoL by demographic and clinical features

The stage of cancer at diagnosis also influenced the QoL score at baseline, with patients diagnosed at earlier stages (In situ and stage I) having higher baseline QoL scores compared to those with more advanced stages. Specifically, patients with stage IV cancer (57.03% of the cohort) had a mean QoL score of 54.81 (95% CI: 52.31, 57.32), significantly lower than those diagnosed in earlier stages, who had a mean of 60 (95% CI: 54.97, 65.02), with a p-value of 0.030. The analysis also revealed that BMI had an impact on QoL, with underweight patients reporting the lowest QoL score at baseline, 44.30(95% CI: 38.56, 50.03), while overweight and obese patients had higher QoL scores 51.44 (95% CI: 49.97, 52.91) and 50.39 (95% CI: 48.70, 52.08), which was statistically significant (*P* = 0.010). Additionally, comorbidities were associated with QoL, with patients having no comorbidities reporting the highest mean QoL score, 54.02(95% CI: 52.35, 55.69), while those with severe comorbidities had the lowest, 43.85 (95% CI: 40.09, 47.61), with a significant p-value of < 0.0001. Regarding treatment modalities, chemoradiation therapy was the most common treatment (44.26%) and was associated with the highest baseline QoL score of 51.03 (95% CI: 49.59, 52.46). This was followed by surgery alone (25.53%) with a QoL score of 50.68 (95% CI: 48.97, 52.38), and radiation therapy alone (6.95%) with a score of 50.47 (95% CI: 46.84, 54.10). Patients who received surgery plus adjuvant chemoradiation therapy (12.60%) had a QoL score of 46.93 (95% CI: 45.38, 50.67), and those who underwent surgery plus adjuvant radiation therapy (10.66%) also had a QoL score of 46.93 (95% CI: 44.38, 49.48). Differences in QoL scores were also observed based on smoking status, with current smokers reporting the lowest QoL scores, 46.88 (95% CI: 45.39, 48.37), and former smokers having higher scores, 51.42(95% CI: 49.89, 52.94, following that never smokers, 53.16 (95% CI: 51.55, 54.78) (*P* < 0.0001). Alcohol consumption was similarly associated with QoL, with never drinkers having a mean QoL score of 49.01 (95% CI: 45.27, 52.75), significantly lower than current drinkers, 51.47(95% CI: 50.30, 52.65) and former drinkers, 47.93(95% CI: 46.37, 49.48), *P* = 0.001 (Table 1).

### QoL over time

**Fig. 1:**
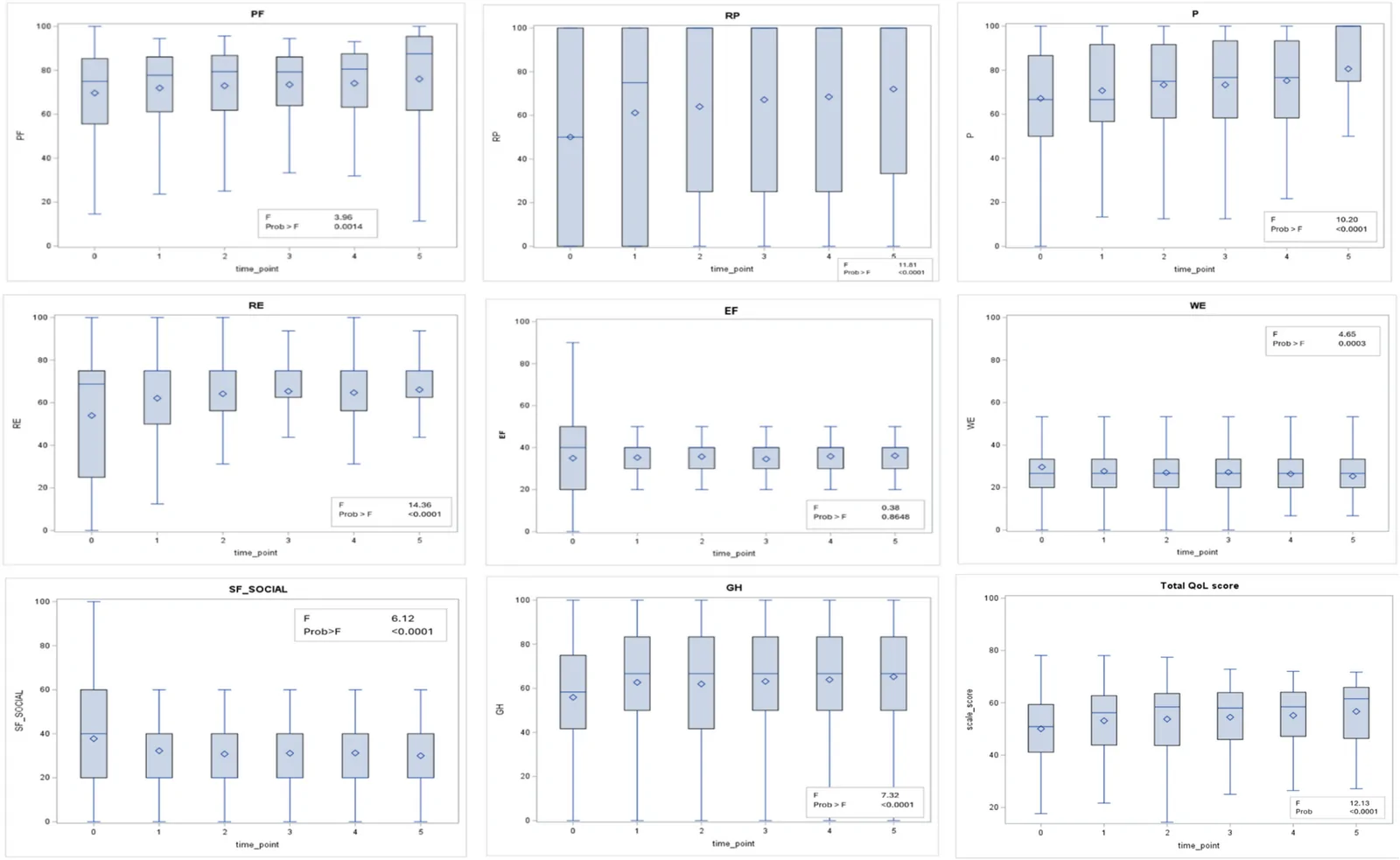
The distribution and trend of various health scores (Total QoL, PF, RP, P, RE, EF, WE, Sf_social, and GH) across six time points (0–5). [physical functioning (PF), role limitations due to physical health (RP), role limitations due to emotional problems (RE), energy/fatigue (EF), emotional well-being (WE), social functioning (SF-Social), pain (P), and general health (GH)]. In the boxplots, the central square line represents the median QoL score for each group, while the diamond represents the mean. In some cases, both a line and a diamond are displayed when the mean and median differ

Significant differences over time were observed in several domains over the course of 5 years (Fig. 1). The physical functioning, role limitations due to emotional problems, and pain domains showed substantial improvements over time (*P* = 0.0014, *P* < 0.0001, and *P* < 0.0001, respectively). In contrast, social functioning consistently worsened across follow-up (*P* < 0.0001), indicating a decline in patients’ social health. Energy/fatigue and emotional well-being remained relatively stable, with non-significant changes at several time points (e.g., *P* = 0.8648 for emotional well-being). Overall, the figure highlights the significant temporal shifts towards improvement in several domains of QoL.

Table 2 illustrates the results of a multiple linear regression analysis assessing the QoL in HNSCC patients over time, stratified by cancer subsite. For the total QoL score, significant early improvements were observed, particularly at the 1-year post-diagnosis [adjusted β: 2.86 (95% CI: 1.56, 4.16)]. Patients with pharyngeal cancer showed the greatest increase in QoL score [adjusted β: 3.56 (95% CI: 1.69, 5.43)], followed by those with oral cavity cancer [adjusted β: 2.56 (95% CI: 0.52, 4.60)]. Laryngeal cancer patients, however, showed no meaningful changes across follow-up [e.g., 1-year adjusted β: 0.93 (95% CI: −2.36, 4.24)], indicating a lack of improvement in overall QoL for this subgroup. (Table 2).

**Table 2:** Multiple linear regression of quality of life and its components over time by HNC subsites

Time point	HNC		Oral cavity		Pharynx		Larynx	
	Adjusted β*	*P*-value	Adjusted β*	*P*-value	Adjusted β*	*P*-value	Adjusted β*	*P*-value
Total Scale score								
Baseline	Reference	-	Reference	-	Reference	-	Reference	-
1 Yr follow up	2.86 (1.56, 4.16)	** < 0.0001**	2.56 (0.52, 4.60)	**0.014**	3.56 (1.69, 5.43)	**0.002**	0.93 (−2.36, 4.24)	0.579
2 Yrs follow up	1.68 (0.96, 2.39)	** < 0.0001**	1.20 (0.01, 2.40)	**0.050**	2.48 (1.49, 3.48)	** < 0.0001**	0.05 (−1.57, 1.68)	0.945
3 Yrs follow up	1.29 (0.76, 1.82)	** < 0.0001**	0.75 (−0.14, 1.65)	0.102	1.87 (1.13, 2.62)	** < 0.0001**	0.35 (−0.95, 1.66)	0.597
4 Yrs follow up	1.10 (0.64, 1.57)	** < 0.0001**	0.54 (−0.23, 1.31)	0.171	1.68 (1.01, 2.35)	** < 0.0001**	0.66 (−0.43, 1.76)	0.237
5 Yrs follow up	1.06 (0.60, 1.52)	** < 0.0001**	0.65 (−0.17, 1.48)	0.125	1.51 (0.89, 2.12)	** < 0.0001**	0.19 (−0.94, 1.32)	0.742
Physical functioning								
1 Yr follow up	1.85 (−0.20, 3.90)	0.077	2.64 (−0.90, 6.19)	0.144	0.41 (−2.43, 3.25)	0.775	−3.76 (−8.46, 0.94)	0.118
2 Yrs follow up	1.26 (0.15, 2.36)	**0.026**	0.83 (−1.17, 2.83)	0.416	1.34 (−0.13, 2.82)	0.075	1.13 (−1.43, 3.70)	0.388
3 Yrs follow up	0.83 (−0.01, 1.67)	0.052	0.96 (−0.56, 2.48)	0.218	0.87 (−0.25, 2.00)	0.128	0.08 (−1.88, 2.06)	0.929
4 Yrs follow up	0.74 (0.01, 1.48)	**0.048**	0.50 (−0.84, 1.84)	0.465	0.77 (−0.24, 1.80)	0.137	0.86 (−0.66, 2.40)	0.269
5 Yrs follow up	0.74 (−0.02, 1.52)	0.059	−0.31 (−1.83, 1.19)	0.680	2.04 (1.09, 2.98)	** < 0.00001**	−1.71 (−3.57, 0.14)	0.073
Role limitations due to physical health								
1 Yr follow up	10.47 (5.47, 15.47)	** < 0.0001**	9.53 (1.05, 18.01)	**0.028**	13.98 (6.85, 21.11)	**0.0001**	−0.22 (−12.13, 11.68)	0.970
2 Yrs follow up	6.48 (3.77, 9.18)	** < 0.0001**	5.29 (0.60, 9.97)	**0.027**	9.25 (5.45, 13.05)	** < 0.0001**	0.20 (−6.05, 6.47)	0.948
3 Yrs follow up	5.22 (3.17, 7.27)	** < 0.0001**	2.69 (−1.01, 6.41)	0.155	7.63 (4.80, 10.47)	** < 0.0001**	1.61 (−3.17, 6.40)	0.510
4 Yrs follow up	4.26 (2.44, 6.07)	** < 0.0001**	2.14 (−1.15, 5.43)	0.203	6.25 (3.68, 8.82)	** < 0.0001**	2.45 (−1.52, 6.42)	0.229
5 Yrs follow up	3.66 (2.09, 5.64)	** < 0.0001**	2.50 (−0.94, 5.96)	0.156	5.49 (3.12, 7.85)	** < 0.0001**	0.97 (−3.09, 5.05)	0.638
Role limitations due to emotional problems								
1 Yr follow up	7.91 (4.79, 11.02)	** < 0.0001**	6.88 (1.50, 12.25)	**0.012**	7.93 (3.54, 12.33)	**0.0004**	8.76 (1.25, 16.27)	**0.023**
2 Yrs follow up	4.95 (3.29, 6.61)	** < 0.0001**	3.76 (0.74, 6.79)	**0.015**	6.16 (3.89, 8.44)	** < 0.0001**	3.47 (−0.33, 7.28)	0.075
3 Yrs follow up	3.54 (2.25, 4.83)	** < 0.0001**	2.29 (−0.09, 4.69)	0.061	4.59 (2.85, 6.34)	** < 0.0001**	2.33 (−0.64, 5.32)	0.126
4 Yrs follow up	2.56 (1.40, 3.72)	** < 0.0001**	2.38 (0.29, 4.48)	**0.026**	2.66 (1.04, 4.27)	**0.001**	2.36 (−0.30, 5.04)	0.085
5 Yrs follow up	2.22 (1.09, 3.34)	**0.0001**	2.39 (0.15, 4.62)	**0.036**	1.98 (0.49, 3.48)	**0.009**	2.48 (−0.11, 5.07)	0.063
Energy/Fatigue								
1 Yr follow up	0.20 (−1.60, 2.00)	0.825	0.12 (−2.90, 3.16)	0.933	1.61 (−1.00, 4.23)	0.226	−3.44 (−7.79, 0.89)	0.121
2 Yrs follow up	0.28 (−0.67, 1.24)	0.557	0.11 (−1.53, 1.75)	0.892	1.23 (−0.13, 2.60)	0.078	−1.92 (−4.21, 0.36)	0.101
3 Yrs follow up	−0.14 (−0.88, 0.59)	0.703	−0.19 (−1.51, 1.13)	0.776	047 (−0.60, 1.55)	0.385	−1.63 (−3.25, −0.01)	0.050
4 Yrs follow up	−0.16 (−0.80, 0.48)	0.626	−0.01 (−1.16, 1.15)	0.988	0.82 (−0.11, 1.76)	0.085	−0.96 (−2.37, 0.43)	0.178
5 Yrs follow up	0.12 (−0.51, 0.76)	0.708	−0.65 (−1.87, 0.56)	0.295	0.57 (−0.30, 1.46)	0.201	−0.03 (−1.53, 1.45)	0.962
Emotional well-being								
1 Yr follow up	−1.85 (−3.35, −0.34)	**0.016**	−1.51 (−4.07, 1.05)	0.248	−1.70 (−3.82, 0.41)	0.115	−2.97 (−6.86, 0.91)	0.136
2 Yrs follow up	−1.29 (−2.11, −0.47)	**0.002**	−1.59 (−3.06, −0.11)	**0.035**	−1.04 (−2.22, 0.12)	0.080	−1.62 (−3.50, 0.26)	0.093
3 Yrs follow up	−0.72 (−1.34, −0.10)	**0.021**	−0.53 (−1.68, 0.61)	0.361	−0.89 (−1.75, −0.04)	0.40	−0.65 (−2.10, 0.78)	0.373
4 Yrs follow up	−0.83 (−1.38, −0.28)	**0.003**	−1.40 (−2.37, −0.42)	**0.005**	−0.68 (−1.46, 0.09)	0.084	−0.27 (−1.57, 1.02)	0.679
5 Yrs follow up	−0.86 (−1.40, −0.32)	**0.001**	−1.21 (−2.25, −0.17)	**0.023**	−0.69 (−1.41, 0.03)	0.063	−1.06 (−2.39, 0.26)	0.119
Social functioning								
1 Yr follow up	−5.15 (−8.4, −2.26)	**0.0005**	−3.61 (−8.55, 1.32)	0.151	−5.76 (−9.93, −1.59)	**0.007**	−5.77 (−12.44, 0.88)	0.090
2 Yrs follow up	−3.23 (−4.78, −1.67)	** < 0.0001**	−1.85 (−4.62, 0.90)	0.189	−4.12 (−6.33, −1.91)	**0.0003**	−3.12 (−6.68, 0.42)	0.086
3 Yrs follow up	−1.99 (−3.20, −0.77)	**0.001**	−0.96 (−3.15, 1.21)	0.385	−3.14 (−4.86, −1.416)	**0.0004**	−0.09 (−2.83, 2.63)	0.944
4 Yrs follow up	−1.53 (−2.60, −0.45)	**0.005**	−0.87 (−2.85, 1.11)	0.390	−2.57 (−4.09, −1.06)	**0.0009**	0.35 (−2.02, 2.73)	0.769
5 Yrs follow up	−1.29 (−2.33, −0.25)	**0.014**	−0.59 (−2.63, 1.43)	0.564	−1.79 (−3.20, −0.39)	**0.012**	−0.87 (−3.26, 1.51)	0.473
Pain								
1 Yr follow up	3.08 (0.69, 5.47)	**0.011**	3.05 (−1.06, 7.16)	0.147	2.54 (−0.78, 5.87)	0.135	3.92 (−1.74, 9.59)	0.176
2 Yrs follow up	2.68 (1.37, 4.00)	** < 0.0001**	2.38 (0.06, 4.70)	**0.044**	2.85 (1.05, 4.64)	**0.002**	2.62 (−0.52, 5.76)	0.104
3 Yrs follow up	1.70 (0.70, 2.70)	**0.0009**	1.00 (−0.79, 2.89)	0.264	2.26 (0.94, 3.58)	**0.0009**	1.00 (−1.39, 3.39)	0.412
4 Yrs follow up	1.62 (0.75, 2.50)	**0.0003**	1.37 (−0.23, 2.97)	0.094	1.95 (0.77, 3.12)	**0.001**	1.56 (−0.43, 3.55)	0.126
5 Yrs follow up	2.08 (1.18, 2.97)	** < 0.0001**	2.59 (0.89, 4.29)	**0.003**	2.28 (1.12, 3.44)	**0.0001**	0.44 (−1.83, 2.71)	0.704
General Health								
1 Yr follow up	6.43 (3.71, 9.14)	** < 0.0001**	4.29 (−0.43, 9.02)	0.076	8.23 (4.48, 11.97)	** < 0.0001**	4.79 (−1.72, 11.31)	0.151
2 Yrs follow up	2.83 (1.33, 4.34)	**0.0002**	1.60 (−1.07, 4.29)	0.241	4.41 (2.35, 6.46)	** < 0.0001**	0.48 (−3.02, 3.99)	0.785
3 Yrs follow up	2.18 (1.03, 3.33)	**0.0002**	1.38 (−0.72, 3.49)	0.198	3.34 (1.76, 4.93)	** < 0.0001**	0.14 (−2.51, 2.80)	0.915
4 Yrs follow up	1.94 (0.95, 2.93)	**0.0001**	1.68 (−0.11, 3.47)	0.067	3.27 (1.87, 4.68)	** < 0.0001**	−0.70 (−2.86, 1.45)	0.524
5 Yrs follow up	1.56 (0.60, 2.51)	**0.001**	0.96 (−0.91, 2.85)	0.313	2.19 (0.93, 3.44)	**0.0007**	0.58 (−1.64, 2.82)	0.606

*Adjusted β for age, gender, *BMI* comorbidity score, modality, smoking, alcohol drinking, cancer type, stage

Physical functioning improved most clearly among pharyngeal cancer patients, particularly at the 5-year follow-up [adjusted β: 2.04 (95% CI: 1.09, 2.98)], while oral cavity and laryngeal cancer patients showed inconsistent patterns. Role limitations due to physical health also improved significantly over time, especially for pharyngeal cancer patients (*p* < 0.0001), whereas emotional well-being tended to worsen, with the greatest decline at 1 year [adjusted β: −1.85 (95% CI: −3.35, −0.34)]. Energy/fatigue showed little change overall, though laryngeal cancer patients exhibited borderline worsening at 3 years [adjusted β: −1.63 (95% CI: −3.25, −0.01)] (Table 2).

Further, the role limitations due to physical health and emotional well-being domains also reflected varying improvements across subsites. Pharyngeal cancer patients showed significant improvements in role limitation due to physical health across all follow-up time points, with adjusted β values ranging from 3.66 to 10.47 for HNSCC combined (*p* < 0.0001), particularly in pharyngeal cancer patients. Similarly, patients with HNSCC showed consistently worsened emotional well-being, with the lowest improvement at 1-year follow-up [adjusted β: −1.85 (95% CI: −3.35, −0.34)]. Regarding energy/fatigue, patients with any HNSCC showed minimal, non-significant energy levels and fatigue improvements across all follow-up periods. Notably, at the 3-year follow-up, patients with laryngeal cancer exhibited a borderline worsening in energy/fatigue, as indicated by an adjusted β of −1.63 (95% CI: −3.25, −0.01) (Table 2).

For role limitations due to emotional problems, significant improvements were observed in the oral cavity and pharyngeal cancer patients, particularly at the 1-year follow-up, with adjusted β values of 6.88 (95% CI: 1.50, 12.25) and 7.93 (95% CI: 3.54, 12.33), respectively, indicating a reduction in emotional distress. However, laryngeal cancer patients showed more variable results, with initial improvement at 1 year (β = 8.76, 95% CI: 1.25, 16.27), but less consistency at later follow-ups, as seen in the 5-year data. All HNSCC patients showed consistent deterioration in Social functioning over time, with the most notable changes observed at the 1-year follow-up [adjusted β: −5.15 (95% CI: −8.4, −2.26)], indicating a substantial decline in Social functioning. Mainly, pharyngeal cancer patients exhibited significantly fewer improvements, particularly at the 1-year follow-up [adjusted β: −5.76 (95% CI: −9.93, −1.59)]. Laryngeal and oral cavity cancer patients showed no significant changes at follow-ups (Table 2).

Pain decreased steadily after diagnosis, with all tumor subsites showing improvement at 1 year [adjusted β: 3.08 (95% CI: 0.69, 5.47)] and sustained relief at 2 years [adjusted β: 2.68 (95% CI: 1.37, 4.00)]. Nonetheless, pain remained an ongoing issue for many patients, and laryngeal cancer patients in particular did not demonstrate consistent improvement [e.g., 1-year β: 3.92 (95% CI: −1.74, 9.59)]. Finally, general health improved over time in all subsites, most notably at the 1-year follow-up [adjusted β: 6.43 (95% CI: 3.71, 9.14)] and remained higher at 5 years [adjusted β: 1.56 (95% CI: 0.60, 2.51)], with pharyngeal cancer patients experiencing the greatest sustained gains.

### Post-diagnosis smoking cessation impact on QoL

**Fig. 2:**
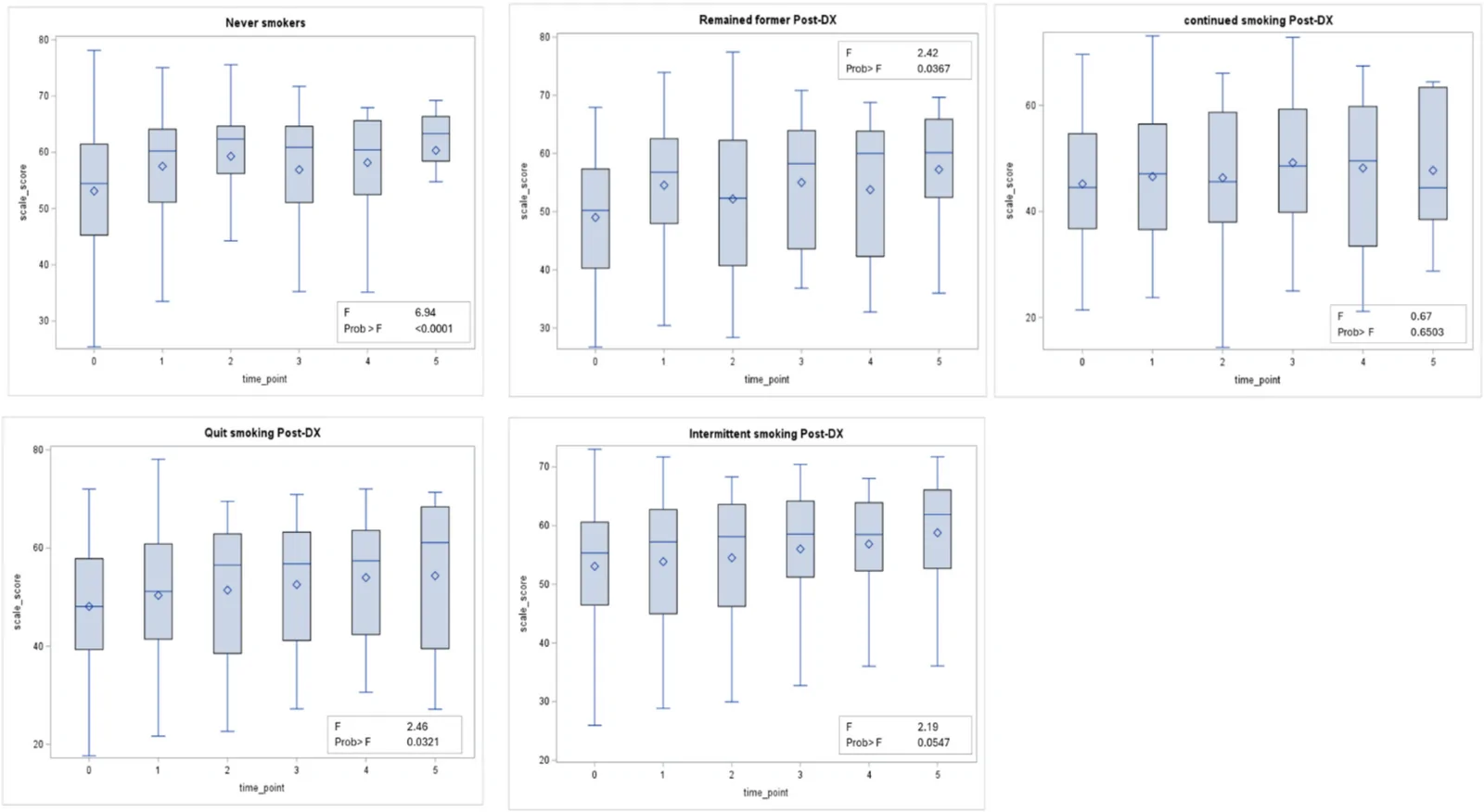
The distribution and trend of overall quality of life score by smoking status after diagnosis. In the boxplots, the central square line represents the median QoL score for each group, while the diamond represents the mean. In some cases, both a line and a diamond are displayed when the mean and median differ

Never smokers and quitting smoking were associated with improved QoL (Table 3; mean QOL scores at baseline, 1 year, and 2 years after diagnosis). For the Total QoL score, patients who had never smoked showed a significant improvement in QoL over time, with mean scores increasing from 53.11 at baseline to 57.51 at 1 year (*P* = 0.01) and further improving to 59.29 at 2 years (*P* < 0.0001). Quitters increased from 48.09 at baseline to 50.35 at 1 year (*P* = 0.11) and 51.42 at 2 years (*P* = 0.04); however, these improvements were less pronounced compared to never-smokers. Continued smokers showed no significant changes in QoL, with scores fluctuating slightly over time (baseline: 45.18, 1 year: 46.57, 2 years: 46.34). Intermittent smokers displayed similar trends to those of continued smokers, with a slight increase in QoL from baseline (53.06) to 1 year (53.85), although these changes were not statistically significant. Significant differences in QoL scores across post-diagnosis smoking groups were observed at all follow-up periods (*P* < 0.0001), indicating that smoking status is a key factor influencing QoL in these patients (Fig. 2).

**Table 3:** Mean score of quality of life and its components at baseline, 1 year and 2 years of follow up by smoking status after diagnosis

	HNC			Oral cavity			Pharynx			Larynx		
Mean score of QoL at Time point	At baseline	1 st Yr	2nd Yr	At baseline	1 st Yr	2nd Yr	At baseline	1 st Yr	2nd Yr	At baseline	1 st Yr	2nd Yr
Total Scale score												
Quit smoking	48.09	**50.35 (*P***^**†**^** = 0.11)**	**51.42 (*P*** **= 0.04)**	50.32	61.36 (P = 0.11)	**54.47 (*P*** **= 0.04)**	49	52.75 (*P* = 0.11)	**54.98 (*P*** ** = 0.02)**	43.75	46.16 (*P* = 0.41)	44.16 (*P* = 0.89)
Never smoker	53.11	**57.51 (*P*** ** = 0.01)**	**59.29 (*P*** ** < 0.0001)**	52.40	**55.50 (*P*** ** = 0.01)**	**56.16 (*P*** ** < 0.0001)**	53.56	**59.24 (*P*** ** = 0.01)**	**61.51 (*P*** ** < 0.0001)**	53.56	53.66 (*P* = 0.98)	55.68 (*P* = 0.54)
Remained former smoker	48.99	**54.54 (*P*** **P = 0.01)**	52.17 (*P* = 0.15)	46.40	**51.86 (*P*** ** = 0.01)**	47.03 (*P* = 0.15)	50.15	**57.03 (*P*** ** = 0.01)**	54.61 (*P* = 0.18)	52.63	48.25 (*P* = 0.61)	52.78 (*P* = 0.97)
Continued smoking	45.18	46.57 (*P* = 0.48)	46.34 (*P* = 0.57)	44.56	45.72 (*P* = 0.49)	45.52 (*P* = 0.57)	47.48	48.77 (*P* = 0.68)	49.51 (*P* = 0.55)	42.80	43.47 (*P* = 0.87)	42.61 (*P* = 0.96)
Intermittent smokesrs	53.06	53.85 (*P* = 0.55)	54.51 (*P* = 0.33)	50.21	53.28 (*P* = 0.55)	51.86 (*P* = 0.33)	54.17	54.43 (*P* = 0.89)	56.32 (*P* = 0.28)	53.81	52.93 (*P* = 0.71)	52.74 (*P* = 0.70)
P-value^‡^	** < 0.0001**	** < 0.0001**	** < 0.0001**	**0.002**	**0.038**	**0.021**	**0.007**	**0.0002**	** < 0.0001**	**0.0006**	**0.105**	**0.008**

^†^ The enclosed p-values, indicating significance between the baseline mean score of quality of life and the score after 1 and 2 years after diagnosis in corresponding smoking group (T-test)

^‡^ The p-value evaluates mean score of quality of life differences across smoking groups (ANOVA test)

In oral cavity cancer patients, never smokers showed significant improvements in QoL, with an increase from 52.40 at baseline to 56.16 at 1 year, 55.50 (*P* = 0.01), and 2 years (*P* < 0.0001). In contrast, quitters exhibited moderate increases in QoL over time, with their scores rising from 50.32 at baseline to 54.47 at the 2-year follow-up (*P* = 0.04). However, patients with continued smoking demonstrated minimal change in QoL, with scores remaining relatively stable and insignificant across follow-up periods. For pharyngeal cancer, never smokers also experienced significant improvements, with QoL increasing from 53.56 at baseline to 59.24 (*P* = 0.01) at 1 year and 61.51 at 2 years (*P* < 0.0001). Like the oral cavity group, quitters showed positive changes, although the improvement was less pronounced, with their QoL scores increasing from 49 at baseline to 54.98 at the 2-year follow-up (*P* = 0.02). Conversely, continued smokers and intermittent smokers exhibited minimal or no significant changes in QoL, suggesting persistent challenges related to smoking status. In the laryngeal cancer patients, never smokers demonstrated the least variation in QoL over time, with scores increasing slightly and insignificant from 47.48 at baseline to 49.51 at 2 years (*P* = 0.55) for continued smokers and 54.17 at baseline to 56.32 at 2 years (*P* = 0.28) for intermittent smokers (Table 3) (Supplement Table 1).

### Changes in voice and dry mouth symptoms across smoking behavior subgroups

**Fig. 3:**
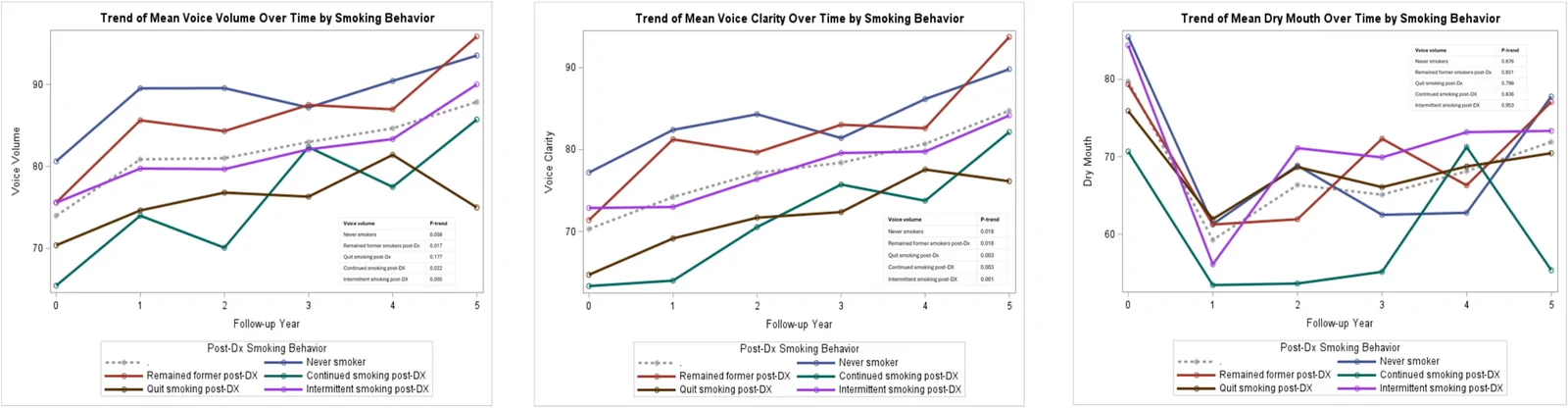
Trends in voice clarity, dry mouth, and voice volume over time by post-diagnosis smoking behavior

Longitudinal analysis revealed distinct patterns in patient-reported outcomes related to voice function and dry mouth across smoking behavior subgroups following cancer diagnosis (Fig. 3). Mean scores for voice volume and voice clarity improved over time among head and neck cancer patients, with notable differences based on post-diagnosis smoking behavior. Participants who remained former smokers or quit smoking after diagnosis consistently exhibited greater improvements in both voice volume and clarity compared to those who continued smoking. Statistically significant linear trends were observed among those who continued smoking, quit smoking, or exhibited intermittent smoking behavior, indicating measurable changes in vocal function over the five-year follow-up period. In contrast, mean dry mouth scores did not show significant longitudinal trends across any smoking behavior subgroup. The patterns for dry mouth were more variable, with no consistent direction of change, suggesting that xerostomia symptoms may be less sensitive to changes in smoking behavior post-diagnosis.

## Discussion

The results of this study suggest that a number of demographic and clinical factors, including cancer subsite, smoking behavior, and treatment modality, could influence long-term QoL in HNSCC patients. Similar to previous studies, patients with HNSCC, particularly oral cavity and pharyngeal cancers, demonstrated significant improvements in QoL over time, particularly in the early years following treatment [8]. However, patients with laryngeal cancer showed no significant improvements in the total score of QoL, aligning with the observations in prior literature where laryngeal cancer patients often experience persistent functional and psychosocial impairments regardless of smoking status [27–29]. Moreover, to our knowledge, this is the first study to demonstrate that post-diagnosis smoking status among HNC patients was associated with differences in QoL scores over time.

The role of smoking in QoL trajectories was evident in the current study, where former and never-smokers indicated significant improvements in QoL while continuing smokers showed no meaningful changes. This finding supports the growing body of literature that emphasizes the negative impact of continued smoking on the health-related QoL of cancer patients, especially in HNC survivors [30–32]. Furthermore, the association between smoking cessation and improvements in QoL in this cohort is consistent with other studies, which have highlighted the benefits of quitting smoking even after a cancer diagnosis [33–35]. Findings also indicated the importance of considering the stage of cancer and BMI in QoL assessments. Patients with advanced-stage disease and those with lower BMI reported poorer baseline QoL scores, with a notable decline observed in patients with stage IV cancer. These findings align with van Nieuwenhuizen et al., who noted that more advanced HNSCC stages were associated with worse QoL outcomes, likely due to the more intensive treatment regimens required, thus supporting tailored interventions for patients facing more severe disease and higher treatment burdens [36–39].

The QoL in HNSCC patients, as demonstrated in this study, varies significantly across tumor subsites and over time following treatment. Consistent with other studies, we found that the overall QoL of HNC patients tended to decline in the early years post-treatment but showed signs of recovery as time progresses [40]. Specifically, patients with pharyngeal tumors showed significant improvements in various QoL domains over the first year, with the most considerable improvements observed in physical functioning and role limitations due to physical health [41]. This aligned with findings from Viganò et al., where the QoL of patients with HNC improved gradually after radiotherapy [42]. In contrast, patients with laryngeal tumors experienced less favorable outcomes, with minimal or no improvements in domains like physical functioning and pain, possibly reflecting the intrinsically greater impact of laryngeal function on QoL compared to pharyngeal function and highlighting the distinct impact of tumor location on QoL.

The findings from this study also emphasize the importance of understanding the psychosocial aspects of QoL in HNSCC patients. The negative psychosocial impact of treatment on QoL, particularly concerning social functioning and emotional distress, has been well documented in previous research [43]. In our study, the most pronounced reductions in social functioning were observed in patients with laryngeal cancer, which was consistent with the literature; such patients experience unique challenges, including swallowing dysfunction and speech impairment, that exacerbate their emotional and social well-being [2]. Future research could focus on innovative interventions, such as the early integration of speech and language therapy, peer support programs tailored to individuals with communication impairments, and technologies that enhance voice rehabilitation or augmentative communication. Additionally, psychosocial interventions specifically designed to address the stigma and isolation associated with vocal loss may help improve quality of life in this population.

Furthermore, the critical role of smoking cessation in improving QoL outcomes was considerable. The significant differences in QoL scores across smoking groups- continuing smokers, former smokers, and never-smokers, suggested that smoking behavior could be a key determinant of patient-reported outcomes, particularly in the early years post-diagnosis. Although no formal cessation program was available at our institution during the study period, our findings, consistent with prior literature and national recommendations, supported the integration of smoking cessation programs as part of the comprehensive care for HNSCC patients to improve long-term QoL [11, 30, 33].

Lastly, the long-term complications of treatment, such as pain and fatigue, add valuable insight into the chronic health issues faced by HNSCC survivors. While some pain relief was observed over time, it remained a significant concern for many patients throughout the follow-up period, particularly laryngeal cancer survivors. This was consistent with the work of Meyer et al., which demonstrated that even after clinical symptoms like aspiration or mucositis have resolved, long-term pain and functional deficits persist, often requiring targeted interventions [3]. These ongoing issues further emphasized the need for integrated pain management strategies to ensure a better QoL for HNSCC survivors, particularly for laryngeal cancer patients [44, 45].

This study has several limitations that should be acknowledged. First, although the follow-up period extended up to eight years, the number of patients with available data after year five was limited, which reduced the precision of estimates in later years. Second, while QoL was assessed prospectively using the validated SF-36 instrument, responses were self-reported and therefore subject to recall and reporting bias. Third, although we adjusted for a wide range of demographic and clinical confounders, residual confounding by unmeasured factors cannot be excluded. Fourth, our cohort was recruited from a single institution, which may limit the generalizability of findings to other settings with different patient populations, treatment protocols, or supportive care resources. Finally, while we demonstrated the importance of smoking status on QoL trajectories, no formal smoking cessation program was available at our institution during the study period, which may have influenced patients’ opportunities to quit and, consequently, their long-term outcomes.

In conclusion, the need for personalized care strategies that account for HNSCC subsite, smoking status, and other prognostic factors to improve the long-term QoL outcomes for HNSCC patients could be crucial. Future studies should focus on interventions targeting modifiable factors, such as smoking cessation and pain management, and to determine their potential to improve survivorship care in this population.

## Supplementary Information

Below is the link to the electronic supplementary material.

Supplementary file1 (DOCX 37 KB)

## Data Availability

No datasets were generated or analysed during the current study.

## References

[1] Johnson DE, Burtness B, Leemans CR, Lui VWY, Bauman JE, Grandis JR. Head and neck squamous cell carcinoma. Nat Rev Dis Primers. 2020;6(1):92. 10.1038/s41572-020-00224-3 PMC7944998 33243986

[2] Zhao L, Moon DH, Avkshtol V, Siropaides CH, Terauchi S, Day AT, et al. Long-term opioid use in patients treated with head and neck intensity-modulated radiotherapy. Support Care Cancer. 2022;30(9):7517–25. 10.1007/s00520-022-07155-7 35666302

[3] Meyer TK, Pisegna JM, Krisciunas GP, Pauloski BR, Langmore SE. Residue influences quality of life independently of penetration and aspiration in head and neck cancer survivors. Laryngoscope. 2017;127(7):1615–21. 10.1002/lary.26387 PMC5423863 27861932

[4] Adamowicz JL, Christensen A, Howren MB, Seaman AT, Kendell ND, Wardyn S, et al. Health-related quality of life in head and neck cancer survivors: evaluating the rural disadvantage. J Rural Health. 2022;38(1):54–62. 10.1111/jrh.12571 PMC8477149 33720456

[5] So WK, Chan RJ, Chan DN, Hughes BG, Chair SY, Choi KC, et al. Quality-of-life among head and neck cancer survivors at one year after treatment–a systematic review. Eur J Cancer. 2012;48(15):2391–408. 10.1016/j.ejca.2012.04.005 22579456

[6] Llewellyn CD, McGurk M, Weinman J. Are psycho-social and behavioural factors related to health related-quality of life in patients with head and neck cancer? A systematic review. Oral Oncol. 2005;41(5):440–54. 10.1016/j.oraloncology.2004.12.006 15878748

[7] Rathod S, Livergant J, Klein J, Witterick I, Ringash J. A systematic review of quality of life in head and neck cancer treated with surgery with or without adjuvant treatment. Oral Oncol. 2015;51(10):888–900. 10.1016/j.oraloncology.2015.07.002 26209066

[8] Vermaire JA, Raaijmakers CPJ, Monninkhof EM, Leemans CR, de Baatenburg Jong RJ, Takes RP, et al. The course of swallowing problems in the first 2 years after diagnosis of head and neck cancer. Support Care Cancer. 2022;30(11):9527–38. 10.1007/s00520-022-07322-w PMC9633522 36040669

[9] von Kroge PR, Bokemeyer F, Ghandili S, Bokemeyer C, Seidel C. The impact of smoking cessation and continuation on recurrence and survival in patients with head and neck cancer: a systematic review of the literature. Oncol Res Treat. 2020;43(10):549–58. 10.1159/000509427 32950990

[10] van Imhoff LC, Kranenburg GG, Macco S, Nijman NL, van Overbeeke EJ, Wegner I, et al. Prognostic value of continued smoking on survival and recurrence rates in patients with head and neck cancer: a systematic review. Head Neck. 2016;38(Suppl 1):E2214–20. 10.1002/hed.24082 25900211

[11] Duffy SA, Terrell JE, Valenstein M, Ronis DL, Copeland LA, Connors M. Effect of smoking, alcohol, and depression on the quality of life of head and neck cancer patients. Gen Hosp Psychiatry. 2002;24(3):140–7. 10.1016/s0163-8343(02)00180-9 12062138

[12] Williams AM, Lindholm J, Cook D, Siddiqui F, Ghanem TA, Chang SS. Association between cognitive function and quality of life in patients with head and neck cancer. JAMA Otolaryngol Head Neck Surg. 2017;143(12):1228–35. 10.1001/jamaoto.2017.2014 PMC5824302 29121151

[13] Ware JE Jr., Gandek B. Overview of the SF-36 health survey and the International Quality of Life Assessment (IQOLA) project. J Clin Epidemiol. 1998;51(11):903–12. 10.1016/s0895-4356(98)00081-x 9817107

[14] Framework IC. The MOS 36-item short-form health survey (SF-36). Med Care. 1992;30(6):473–83. 1593914

[15] De Graeff A, De Leeuw J, Ros W, Hordijk G, Blijham G, Winnubst J. A prospective study on quality of life of patients with cancer of the oral cavity or oropharynx treated with surgery with or without radiotherapy. Oral Oncol. 1999;35(1):27–32. 10.1016/s1368-8375(98)00049-9 10211307

[16] Ogura K, Bartelstein MK, Yakoub MA, Nikolic Z, Boland PJ, Healey JH. Minimal clinically important differences in SF-36 global score: current value in orthopedic oncology. J Orthop Res. 2021;39(10):2116–23. 10.1002/jor.24944 PMC9348556 33617087

[17] Ogura K, Yakoub MA, Christ AB, Fujiwara T, Nikolic Z, Boland PJ, et al. What are the minimum clinically important differences in SF-36 scores in patients with orthopaedic oncologic conditions? Clin Orthop Relat Res. 2020;478(9):2148–58. 10.1097/CORR.0000000000001341 PMC7431256 32568896

[18] Kull CE, Vifian L, Leuppi JD, Baumgartner C, Bösing M, Roser K, et al. A prospective cohort study on the trajectory of health-related quality of life in adult childhood cancer survivors attending a follow-up care program. Sci Rep. 2025;15(1):8332. 10.1038/s41598-025-92820-0 PMC11894196 40065016

[19] Bjorner JB, Wolden ML, Gundgaard J, Miller KA. Benchmarks for interpretation of score differences on the SF-36 health survey for patients with diabetes. Value Health. 2013;16(6):993–1000. 10.1016/j.jval.2013.06.022 24041349

[20] Grönkvist R, Vixner L, Äng B, Grimby-Ekman A. Measurement error, minimal detectable change, and minimal clinically important difference of the short form-36 health survey, hospital anxiety and depression scale, and pain numeric rating scale in patients with chronic pain. J Pain. 2024;25(9):104559. 10.1016/j.jpain.2024.104559 38734041

[21] Mouelhi Y, Jouve E, Castelli C, Gentile S. How is the minimal clinically important difference established in health-related quality of life instruments? Review of anchors and methods. Health Qual Life Outcomes. 2020;18:1–17. 10.1186/s12955-020-01344-w PMC7218583 32398083

[22] Fleming ST, Sabatino SA, Kimmick G, Cress R, Wu X-C, Trentham-Dietz A, et al. Developing a claim-based version of the ACE-27 comorbidity index: a comparison with medical record review. Med Care. 2011;49(8):752–60. 10.1097/MLR.0b013e318215d7dd 21490514

[23] Zhu X. Comparison of four methods for handing missing data in longitudinal data analysis through a simulation study. Open J Stat. 2014;4(11):933–44.

[24] Rogers SN, Gwanne S, Lowe D, Humphris G, Yueh B, Weymuller EA Jr. The addition of mood and anxiety domains to the University of Washington quality of life scale. Head Neck. 2002;24(6):521–9. 10.1002/hed.10106 12112548

[25] Datema FR, Ferrier MB, van der Schroeff MP, de Baatenburg Jong RJ. Impact of comorbidity on short-term mortality and overall survival of head and neck cancer patients. Head Neck: J Sci Specialties Head Neck. 2010;32(6):728–36. 10.1002/hed.21245 19827120

[26] Zhou T, Huang W, Wang X, Zhang J, Zhou E, Tu Y, et al. Global burden of head and neck cancers from 1990 to 2019. iScience. 2024. 10.1016/j.isci.2024.109282. PMC10918270 38455975

[27] Miller MC, Shuman AG. Survivorship in head and neck cancer: a primer. JAMA Otolaryngol Head Neck Surg. 2016;142(10):1002–8. 10.1001/jamaoto.2016.1615 27414519

[28] Midgley AW, Lowe D, Levy AR, Mepani V, Rogers SN. Exercise program design considerations for head and neck cancer survivors. Eur Arch Otorhinolaryngol. 2018;275(1):169–79. 10.1007/s00405-017-4760-z PMC5754417 29058083

[29] Kemps GJF, Krebbers I, Pilz W, Vanbelle S, Baijens LWJ. Affective symptoms and swallow-specific quality of life in total laryngectomy patients. Head Neck. 2020;42(11):3179–87. 10.1002/hed.26365 PMC7586820 32621568

[30] Jensen K, Jensen AB, Grau C. Smoking has a negative impact upon health related quality of life after treatment for head and neck cancer. Oral Oncol. 2007;43(2):187–92. 10.1016/j.oraloncology.2006.02.006 16860590

[31] Halms T, Strasser M, Hasan A, Rüther T, Trepel M, Raab S, et al. Smoking and quality of life in lung cancer patients: systematic review. BMJ Support Palliat Care. 2024;13(e3):e686–94. 10.1136/spcare-2023-004256 37607808

[32] Caini S, Del Riccio M, Vettori V, D’Ecclesiis O, Bonomo P, Locatello LG, et al. Post-diagnosis smoking cessation and survival of patients with head and neck cancer: a systematic review and meta-analysis. Br J Cancer. 2022;127(11):1907–15. 10.1038/s41416-022-01945-w PMC9681856 35999273

[33] Martínez Ú, Brandon KO, Sutton SK, Brandon TH, Simmons VN. Does smoking abstinence predict cancer patients’ quality of life over time? Psychooncology. 2019;28(8):1702–11. 10.1002/pon.5145 PMC6691965 31212391

[34] Doerr F, Leschczyk T, Grapatsas K, Menghesha H, Baldes N, Schlachtenberger G, et al. Postoperative tobacco cessation improves quality of life, lung function and long-term survival in non-small-cell lung cancer patients. Cancers (Basel). 2024. 10.3390/cancers16020465. PMC10813915 38275905

[35] Lowy DR, Fiore MC, Willis G, Mangold KN, Bloch MH, Baker TB. Treating smoking in cancer patients: an essential component of cancer care-the new National Cancer Institute tobacco control monograph. JCO Oncol Pract. 2022;18(12):e1971–6. 10.1200/OP.22.00385 PMC10166433 36343305

[36] Danson SJ, Rowland C, Rowe R, Ellis S, Crabtree C, Horsman JM, et al. The relationship between smoking and quality of life in advanced lung cancer patients: a prospective longitudinal study. Support Care Cancer. 2016;24(4):1507–16. 10.1007/s00520-015-2928-x 26364190

[37] van Nieuwenhuizen AJ, Buffart LM, Brug J, Leemans CR, Verdonck-de Leeuw IM. The association between health related quality of life and survival in patients with head and neck cancer: a systematic review. Oral Oncol. 2015;51(1):1–11. 10.1016/j.oraloncology.2014.09.002 25262163

[38] Hammerlid E, Bjordal K, Ahlner-Elmqvist M, Boysen M, Evensen JF, Biörklund A, et al. A prospective study of quality of life in head and neck cancer patients. Part I: at diagnosis. Laryngoscope. 2001;111(4 Pt 1):669–80. 10.1097/00005537-200104000-00021 11359139

[39] List MA, Siston A, Haraf D, Schumm P, Kies M, Stenson K, et al. Quality of life and performance in advanced head and neck cancer patients on concomitant chemoradiotherapy: a prospective examination. J Clin Oncol. 1999;17(3):1020–8. 10.1200/JCO.1999.17.3.1020 10071297

[40] Verdonck-de Leeuw IM, Buffart LM, Heymans MW, Rietveld DH, Doornaert P, de Bree R, et al. The course of health-related quality of life in head and neck cancer patients treated with chemoradiation: a prospective cohort study. Radiother Oncol. 2014;110(3):422–8. 10.1016/j.radonc.2014.01.002 24582144

[41] Bjordal K, Ahlner-Elmqvist M, Hammerlid E, Boysen M, Evensen JF, Biörklund A, et al. A prospective study of quality of life in head and neck cancer patients. Part II: longitudinal data. Laryngoscope. 2001;111(8):1440–52. 10.1097/00005537-200108000-00022 11568582

[42] Viganò A, De Felice F, Iacovelli NA, Alterio D, Ingargiola R, Casbarra A, et al. Quality of life changes over time and predictors in a large head and neck patients’ cohort: secondary analysis from an Italian multi-center longitudinal, prospective, observational study-a study of the Italian Association of Radiotherapy and Clinical Oncology (AIRO) head and neck working group. Support Care Cancer. 2023;31(4):220. 10.1007/s00520-023-07661-2 PMC10023607 36930353

[43] McQuellon RP, Hurt GJ. The psychosocial impact of the diagnosis and treatment of laryngeal cancer. Otolaryngol Clin North Am. 1997;30(2):231–41. 9052667

[44] Burgin SJM, Spector ME, Pearson AT, Bellile E, Vainshtein JM, Rosko A, et al. Long-term neck and shoulder function among survivors of oropharyngeal squamous cell carcinoma treated with chemoradiation as assessed with the neck dissection impairment index. Head Neck. 2021;43(5):1621–8. 10.1002/hed.26636 PMC13174022 33580560

[45] Brook I. Early side effects of radiation treatment for head and neck cancer. Cancer Radiother. 2021;25(5):507–13. 10.1016/j.canrad.2021.02.001 33685809

