# Supplementary material for: Long-term quality of life in head and neck cancer: the role of postdiagnosis smoking behavior
Source: J Cancer Surviv. 2025 Oct 21;20(5):2106–18. doi: 10.1007/s11764-025-01894-2 (PMC13723569; doi:10.1007/s11764-025-01894-2)
Supplement: Supplementary file 1 [file 11764_2025_1894_MOESM1_ESM.docx]

| Supplement Table 1- Mean score of quality of life and its components at baseline, 1 year and 2 years of follow up by smoking status after diagnosis. | | | | | | | | | | | | |
| --- | --- | --- | --- | --- | --- | --- | --- | --- | --- | --- | --- | --- |
|  | **HNC** | | | **Oral cavity** | | | **Pharynx** | | | **Larynx** | | |
| Mean score of QoL at Time point | **At baseline** | **1^st^ Year** | **2^nd^ Year** | **At baseline** | **1^st^ Year** | **2^nd^ Year** | **At baseline** | **1^st^ Year** | **2^nd^ Year** | **At baseline** | **1^st^ Year** | **2^nd^ Year** |
| Total Scale score |  |  |  |  |  |  |  |  |  |  |  |  |
| Quit smoking | 48.09 | **50.35 (P^†^=0.11)** | **51.42 (P=0.04)** | 50.32 | 61.36 (P=0.11) | **54.47 (P=0.04)** | 49 | 52.75 (P=0.11) | **54.98 (P=0.02)** | 43.75 | 46.16 (P=0.41) | 44.16 (P=0.89) |
| Never smoker | 53.11 | **57.51 (P= 0.01)** | **59.29**  **(P <0.0001)** | 52.40 | **55.50 (P=0.01)** | **56.16**  **(P<0.0001)** | 53.56 | **59.24 (P=0.01)** | **61.51 (P<0.0001)** | 53.56 | 53.66 (P=0.98) | 55.68 (P=0.54) |
| Remained former smoker | 48.99 | **54.54 (P=0.01)** | 52.17 (P=0.15) | 46.40 | **51.86 (P=0.01)** | 47.03 (P=0.15) | 50.15 | **57.03 (P=0.01)** | 54.61 (P=0.18) | 52.63 | 48.25 (P=0.61) | 52.78 (P=0.97) |
| Continued smoking | 45.18 | 46.57 (P=0.48) | 46.34 (P=0.57) | 44.56 | 45.72 (P=0.49) | 45.52 (P=0.57) | 47.48 | 48.77 (P=0.68) | 49.51 (P=0.55) | 42.80 | 43.47 (P=0.87) | 42.61 (P=0.96) |
| Intermittent smokers | 53.06 | 53.85 (P=0.55) | 54.51 (P=0.33) | 50.21 | 53.28 (P=0.55) | 51.86 (P=0.33) | 54.17 | 54.43 (P=0.89) | 56.32 (P=0.28) | 53.81 | 52.93 (P=0.71) | 52.74 (P=0.70) |
| P-value^‡^ | **<0.0001** | **<0.0001** | **<0.0001** | **0.002** | **0.038** | **0.021** | **0.007** | **0.0002** | **<0.0001** | **0.0006** | **0.105** | **0.008** |
| Physical functioning |  |  |  |  |  |  |  |  |  |  |  |  |
| Quit smoking | 66.26 | 67.62 (P= 0.54) | 70.50 (P= 0.09) | 66.33 | 67.58 (P= 0.72) | 71.12 (P= 0.21) | 69.11 | 68.87 (P= 0.95) | 75.10 (P= 0.15) | 62.35 | 66.08 (P= 0.84) | 64.40 (P= 0.66) |
| Never smoker | 75.71 | 77.81 (P= 0.28) | **80.31 (P=0.01)** | 70.86 | 74.10 (P= 0.34) | 75.12 (P= 0.21) | 78.81 | 80.38 (P= 0.50) | **82.99 (P= 0.05)** | 79.34 | 77.25 (P= 0.82) | 83.24 (P= 0.57) |
| Remained former smoker | 66.94 | **74 (P= 0.03**) | 69.68 (P= 0.43) | 63.74 | 72.27 (P= 0.10) | 61.86 (P= 0.75) | 68.14 | 74.99 (P= 0.16) | 72.26 (P= 0.41) | 72.22 | 73.95 (P= 0.88) | 73.71 (P= 0.85) |
| Continued smoking | 59.86 | 64.42 (P= 0.14) | 62.56 (P=0.42) | 57.83 | 60.57 (P= 0.62) | 58.75 (P= 0.89) | 64.85 | 66.96 (P= 0.68) | 66.01 (P= 0.82) | 56.18 | 64.17 (P= 0.15) | 61.88 (P=0.33) |
| Intermittent smokers | 75.55 | 72.85 (P=0.20) | 74.39 (P= 0.62) | 71.14 | 70.58 (P= 0.90) | 69.34 (P= 0.72) | 76.33 | 72.09 (P= 0.13) | 75.35 (P= 0.75) | 79.79 | 79.16 (P= 0.85) | 78.37 (P= 0.75) |
| P-value | **<0.0001** | **<0.0001** | **<0.0001** | **0.004** | 0.110 | **0.022** | **<0.0001** | **0.001** | **0.0003** | **<0.0001** | **0.022** |  |
| Role limitations due to physical health |  |  |  |  |  |  |  |  |  |  |  |  |
| Quit smoking | 45.77 | 50.70 (P= 0.34) | 54.59 (P= 0.15) | 50.44 | 51.88 (P= 0.74) | 65.62 (P= 0.08) | 48.69 | 60.93 (P= 0.58) | 65.62 (P= 0.48) | 35.25 | 36.87 (P= 0.86) | 32.40 (P= 0.78) |
| Never smoker | 55.70 | **75.18 (P<0.0001)** | **83.55 (P<0.0001)** | 57.93 | 71.07 (P= 0.86) | 88.63 (P<0.0001) | 55.21 | **79.22 (P= 0.01)** | **88.63 (P<0.0001)** | 43.75 | 62.50 (P= 0.46) | 71.42 (P= 0.55) |
| Remained former smoker | 43.75 | **62.17 (P= 0.03)** | 56.39 (P= 0.13) | 30.88 | 45.83 (P= 0.34) | 65.21 (P= 0.15) | 48.68 | 72.82 (P= 0.49) | 65.21 (P= 0.54) | 64.58 | 50 (P= 0.58) | 56.25 (P= 0.69) |
| Continued smoking | 35.49 | 41.34 (P= 0.41) | 40.45 (P= 0.47) | 36.38 | 40 (P= 0.55) | 47.72 (P= 0.55) | 40.54 | 46.87 (P= 087) | 47.72 (P= 0.62) | 26.92 | 32.69 (P= 0.68) | 28.12 (P= 0.92) |
| Intermittent smokers | 63.73 | 65.25 (P= 0.76) | 67.04 (P= 0.56) | 56.61 | 67.76 (P= 0.86) | 71.93 (P= 0.47) | 66.66 | 67 (P= 0.55) | 71.93 (P= 0.86) | 65.21 | 55.43 (P= 0.40) | 64.70 (P= 0.97) |
| P-value | **<0.0001** | **<0.0001** | **<0.0001** | **0.014** | **0.028** | **0.0002** | **0.025** | **0.006** | **0.0002** | **0.007** | 0.296 | 0.035 |
| † The enclosed p-values, indicating significance between the baseline mean score of quality of life and the score after 1 and 2 years after diagnosis in corresponding smoking group (T-test).  ‡ The p-value evaluates mean score of quality of life differences across smoking groups (ANOVA test). | | | | | | | | | | | | |

| Cont. Supplement Table 1- Mean score of quality of life and its components at baseline, 1 year and 2 years of follow up by smoking status after diagnosis. | | | | | | | | | | | | |
| --- | --- | --- | --- | --- | --- | --- | --- | --- | --- | --- | --- | --- |
|  | **HNC** | | | **Oral cavity** | | | **Pharynx** | | | **Larynx** | | |
| Mean score of QoL at Time point | **At baseline** | **1^st^ Year** | **2^nd^ Year** | **At baseline** | **1^st^ Year** | **2^nd^ Year** | **At baseline** | **1^st^ Year** | **2^nd^ Year** | **At baseline** | **1^st^ Year** | **2^nd^ Year** |
| Role limitations due to emotional problems |  |  |  |  |  |  |  |  |  |  |  |  |
| Quit smoking | 50.9 | 59.92 (P= 0.20) | 61.60 (P= 0.30) | 49.33 | 59.51(P= 0.20) | 64.35 (P= 0.20) | 54.16 | 61.66 (P= 0.33) | 66.40 (P= 0.34) | 45.88 | 58.28 (P= 0.75) | 53.16 (P= 0.70) |
| Never smoker | 57.67 | **64.46 (P= 0.03)** | **68.78 (P<0.0001)** | 61.90 | 64.42 (P= 0.84) | 67.59 (P= 0.22) | 54.05 | **63.71 (P= 0.02)** | **70.64 (P<0.0001)** | 65.62 | 71.87 (P= 0.58) | 57.73 (P= 0.82) |
| Remained former smoker | 56.62 | 65.78 (P= 0.21) | **64.34 (P= 0.03)** | 50.18 | 62.98 (P= 0.95) | 52.08 (P= 0.96) | 59.86 | **69.02 (P= 0.04)** | **69.27 (P=0.01)** | 64.58 | 56.25 (P= 0.35) | 67.96 (P= 0.20) |
| Continued smoking | 45.63 | 48.43 (P= 0.75) | 53.16 (P= 0.56) | 45.52 | 46.66 (P= 0.58) | 47.68 (P= 0.89) | 50.33 | 50.26 (P= 0.76) | 57.10 (P= 0.79) | 39.35 | 47.11 (P= 0.98) | 53.90 (P= 0.71) |
| Intermittent smokers | 58.81 | **66.14 (P=0.04)** | **67.82 (P= 0.01)** | 59.28 | 66.02 (P= 0.23) | 69.02 (P= 0.10) | 56.36 | 64.83 (P= 0.31) | 66.62 (P= 0.14) | 65.76 | 70.65 (P= 0.10) | 69.73 (P= 0.37) |
| P-value | **0.0008** | **0.0002** | **0.0003** | **0.022** | 0.128 | **0.014** | 0.652 | 0.065 | 0.108 | 0.001 | 0.075 | 0.194 |
| Energy/Fatigue |  |  |  |  |  |  |  |  |  |  |  |  |
| Quit smoking | 33.28 | 34.50 (P=0.49) | 35.28 (P=0.32) | 33.21 | 34.61 (P=0.60) | 37.50 (P=0.19) | 33.92 | 35.20 (P=0.68) | 36.56 (P=0.44) | 32.56 | 33.50 (P=0.79) | 31.48 (P=0.78) |
| Never smoker | 35.12 | 36.25 (P=0.53) | 37.27 (P=0.24) | 34.12 | 33.07 (P=0.72) | 34.73 (P=0.83) | 35.60 | 38.42 (P=0.25) | 38.18 (P=0.31) | 37.50 | 36.25 (P=0.88) | 42.85 (P=0.57) |
| Remained former smoker | 35.18 | **41.25 (P=0.02)** | 38.40 (P=0.24) | 35.29 | 41.53 (P=0.18) | 35 (P=0.95) | 33.78 | **42.17 (P=0.02)** | 40.83 (P=0.08) | 39.16 | 35 (P=0.66) | 36.25 (P=0.66) |
| Continued smoking | 35.96 | 33.26 (P=0.33) | 33.33 (P=0.30) | 33.21 | 36.66 (P=0.72) | 37.50 (P=0.71) | 34.86 | 33.33 (P=0.74) | 34.34 (P=0.90) | 39.23 | 29.23 (P=0.09) | **28.12 (P=0.02)** |
| Intermittent smokers | 35.69 | 34.01 (P=0.36) | 34.33 (P=0.51) | 34.28 | 33.07 (P=0.76) | 30 (P=0.32) | 36.66 | 36 (P=0.78) | 38 (P=0.61) | 34.78 | 29.13 (P=0.16) | 29.41 (P=0.27) |
| P-value | 0.6829 | **0.0525** | 0.2192 | 0.9767 | 0.4235 | 0.3527 | 0.8763 | 0.1604 | 0.5355 | 0.4846 | 0.6912 | 0.2143 |
| Emotional well-being |  |  |  |  |  |  |  |  |  |  |  |  |
| Quit smoking | 28.90 | 28.59 (P=0.12) | 26.89 (P=0.19) | 30.83 | 29.87 (P=0.65) | 28.57 (P=0.44) | 26.01 | 27.33 (P=0.58) | 25.41 (P=0.79) | 29.91 | 28.50 (P=0.65) | 26.91 (P=0.30) |
| Never smoker | 29.44 | 27 (P=0.20) | **26.12 (P=0.02)** | 27.88 | 26.28 (P=0.12) | **23.33 (P=0.03)** | 30.03 | 27.89 (P=0.26) | 27.89 (P=0.27) | 35 | 23.33 (P=0.12) | 24.76 (P=0.20) |
| Remained former smoker | 28.91 | 30.16 (P=0.91) | 25.90 (P=0.33) | 29.80 | 31.28 (P=0.79) | 27.22 (P=0.73) | 29.90 | 30.43 (P=0.87) | 25.83 (P=0.33) | 23.33 | 25 (P=0.79) | 24.16 (P=0.85) |
| Continued smoking | 33.46 | 31.98 (P=0.58) | 31.34 (P=0.38) | 32.19 | 33.11 (P=0.82) | 30.74 (P=0.71) | 33.51 | 30.27 (P=0.40) | 31.59 (P=0.65) | 35.38 | 33.84 (P=0.82) | 31.66 (P=0.39) |
| Intermittent smokers | 28.90 | **25.01 (P=0.03)** | 25.92 (P=0.17) | 26.66 | 23.93 (P=0.11) | 24.34 (P=0.47) | 29.90 | 27.02 (P=0.14) | 27.73 (P=0.30) | 24.63 | 20.28 (P=0.11) | 22.74 (P=0.58) |
| P-value | **0.0342** | **0.0029** | **0.0551** | 0.3269 | **0.0484** | 0.2505 | 0.1569 | 0.5933 | 0.3577 | **0.0319** | **0.0452** | 0.1663 |
| † The enclosed p-values, indicating significance between the baseline mean score of quality of life and the score after 1 and 2 years after diagnosis in corresponding smoking group (T-test).  ‡ The p-value evaluates mean score of quality of life differences across smoking groups (ANOVA test). | | | | | | | | | | | | |

| Cont. Supplement Table 1- Mean score of quality of life and its components at baseline, 1 year and 2 years of follow up by smoking status after diagnosis. | | | | | | | | | | | | |
| --- | --- | --- | --- | --- | --- | --- | --- | --- | --- | --- | --- | --- |
|  | **HNC** | | | **Oral cavity** | | | **Pharynx** | | | **Larynx** | | |
| Mean score of QoL at Time point | **At baseline** | **1^st^ Year** | **2^nd^ Year** | **At baseline** | **1^st^ Year** | **2^nd^ Year** | **At baseline** | **1^st^ Year** | **2^nd^ Year** | **At baseline** | **1^st^ Year** | **2^nd^ Year** |
| Social functioning |  |  |  |  |  |  |  |  |  |  |  |  |
| Quit smoking | 40.27 | **34.08 (P=0.04)** | **31.26 (P=0.006)** | 38.92 | 32.69 (P=0.20) | 30 (P=0.07) | 39.60 | 35.60 (P=0.48) | **28.12 (P=0.04)** | 43.07 | 34 (P=0.08) | 36.29 (P=0.27) |
| Never smoker | 35.18 | **29.33 (P=0.03)** | **24.90 (P=0.001)** | 34.60 | 32.94 (P=0.72) | 26.31 (P=0.06) | 34.94 | **26.57 (P=.01)** | **23.07 (P=0.001)** | 42.50 | 32.50 (P=0.31) | 34.28 (P=0.42) |
| Remained former smoker | 36.86 | **27.50 (P=0.03)** | **27.27 (P=0.02)** | 38.23 | 27.69 (0.12) | 30 (P=0.32) | 38.37 | 26.95 (P=0.09) | 28.33 (P=0.09) | 28.33 | 30 (P=0.85) | 20 (P=0.34) |
| Continued smoking | 45.76 | 41.92 (P=0.39) | 45 (P=0.86) | 43.41 | 41.33 (P=0.80) | 47.77 (P=0.58) | 44.32 | 42.50 (P=0.79) | 40 (P=0.56) | 51.53 | 41.53 (P=0.28) | 49.33 (P=0.77) |
| Intermittent smokers | 32.30 | 30.94 (P=0.65) | 30.44 (P=0.58) | 29.14 | 29.23 (P=0.98) | 33.91 (P=0.48) | 35 | 31.46 (P=0.39) | 31.60 (P=0.47) | 28.69) | 32.17 (P=0.61) | 22.35 (P=0.36) |
| P-value | **0.0011** | **0.0103** | **<0.0001** | 0.1789 | 0.5205 | **0.0215** | 0.3579 | **0.0411** | **0.0412** | **0.0122** | 0.7821 | **0.0048** |
| Pain |  |  |  |  |  |  |  |  |  |  |  |  |
| Quit smoking | 65.54 | 67.28 (P=0.52) | 71.13 (P=0.07) | 69.27 | 68.96 (P=0.94) | 77.97 (P=0.09) | 67.48 | 69.17 (P=0.71) | 73.07 (P=0.28) | 57.60 | 62.70 (P=0.31) | 61.75 (P=0.45) |
| Never smoker | 72.92 | **77.62 (P=0.05)** | **81.50 (P=0.001)** | 68.07 | 74.64 (P=0.12) | 76.29 (P=0.07) | 76.08 | 80.17 (P=0.19) | **84.79 (P=0.007)** | 75.83 | 72.50 (P=0.72) | 78.80 (P=0.78) |
| Remained former smoker | 64.76 | 68.04 (P=0.42) | **73.93 (P=0.02**) | 59.50 | 60.25 (P=0.89) | 62.77 (P=0.56) | 68.94 | 72.82 (P=0.51) | 75.62 (P=0.25) | 66.38 | 65.83 (P=0.96) | **85.62 (P=0.03)** |
| Continued smoking | 57.55 | 59.10 (P=0.66) | 58.28 (P=0.84) | 56.82 | 54.66 (P=0.67) | 51.43(P=0.37) | 62.36 | 64.09 (P=0.79) | 66.88 (P=0.47) | 52.09 | 55 (P=0.63) | 53.64 (P=0.82) |
| Intermittent smokers | 71.26 | 72.67 (P=0.57) | 74.61 (P=0.23) | 67.23 | 71.04 (P=0.45) | 72.17(P=0.36) | 73.05 | 73.66 (P=0.85) | 75.65 (P=0.51) | 71.81 | 72.17 (P=0.93) | 74.80 (P=0.60) |
| P-value | **<0.0001** | **<0.0001** | **<0.0001** | **0.0296** | **0.0116** | **0.0002** | **0.0177** | **0.0065** | **0.0047** | **0.0032** | 0.0879 | **0.0015** |
| General Health |  |  |  |  |  |  |  |  |  |  |  |  |
| Quit smoking | 53.65 | **58.82 (P=0.07)** | **61.59 (P=0.02)** | 60.90 | 64.07 (P=0.46) | 66.66 (P=0.27) | 53.68 | 60.78 (P=0.12) | **69.53 (P=0.004)** | 43.37 | 49.37 (P=0.34) | 46.91 (P=0.62) |
| Never smoker | 62.26 | **69.70 (P=0.004)** | **72.56 (P=0.0002)** | 61.84 | 65.54 (P=0.42) | 68.96 (P=0.15) | 63.73 | **74.24 (P=0.001)** | **76.76 (P=<0.0001)** | 48.95 | 53.12 (P=0.56) | 52.38 (P=0.73) |
| Remained former smoker | 56.80 | **65.20 (P=0.04)** | 60.41 (P=0.43) | 60.10 | 66.66 (P=0.29) | 68.05 (P=0.25) | 52.02 | **67.02 (P=0.01)** | 57.29 (P=0.46) | 62.50 | 50 (P=0.27) | 58.33 (P=0.68) |
| Continued smoking | 45.51 | 52.08 (P=0.12) | 45.38 (P=0.97) | 47.42 | 52.77 (P=0.56) | 44.11 (P=0.71) | 49.09 | 55.90 (P=0.27) | 53.62 (P=0.42) | 37.65 | 44.23 (P=0.31) | 34.89 (P=0.66) |
| Intermittent smokers | 58.46 | 63.04 (P=0.09) | 60.34 (P=0.56) | 56.19 | 61.53 (P=0.36) | 56.15 (P=0.99) | 59.14 | 63.38 (P=0.25) | 62.41 (P=0.46) | 59.78 | 64.49 (P=0.39) | 59.80 (P=0.99) |
| P-value | **<0.0001** | **<0.0001** | **<0.0001** | **0.0239** | 0.4789 | **0.0081** | **0.0058** | **0.0006** | **<0.0001** | **0.0020** | 0.1024 | 0.0627 |
| † The enclosed p-values, indicating significance between the baseline mean score of quality of life and the score after 1 and 2 years after diagnosis in corresponding smoking group (T-test).  ‡ The p-value evaluates mean score of quality of life differences across smoking groups (ANOVA test). | | | | | | | | | | | | |
